# Influenza viral membrane fusion is sensitive to sterol concentration but surprisingly robust to sterol chemical identity

**DOI:** 10.1038/srep29842

**Published:** 2016-07-19

**Authors:** Katarzyna E. Zawada, Dominik Wrona, Robert J. Rawle, Peter M. Kasson

**Affiliations:** 1 Departments of Molecular Physiology and Biological Physics and of Biomedical Engineering, University of Virginia, Charlottesville, VA 22908, United States

## Abstract

Influenza virions are enriched in cholesterol relative to the plasma membrane from which they bud. Previous work has shown that fusion between influenza virus and synthetic liposomes is sensitive to the amount of cholesterol in either the virus or the target membrane. Here, we test the chemical properties of cholesterol required to promote influenza fusion by replacing cholesterol with other sterols and assaying viral fusion kinetics. We find that influenza fusion with liposomes is surprisingly robust to sterol chemical identity, showing no significant dependence on sterol identity in target membranes for any of the sterols tested. In the viral membrane, lanosterol slowed fusion somewhat, while polar sterols produced a more pronounced slowing and inhibition of fusion. No other sterols tested showed a significant perturbation in fusion rates, including ones previously shown to alter membrane bending moduli or phase behavior. Although fusion rates depend on viral cholesterol, they thus do not require cholesterol’s ability to support liquid-liquid phase coexistence. Using electron cryo-microscopy, we further find that sterol-dependent changes to hemagglutinin spatial patterning in the viral membrane do not require liquid-liquid phase coexistence. We therefore speculate that local sterol-hemagglutinin interactions in the viral envelope may control the rate-limiting step of fusion.

Fusion between the influenza viral envelope and host cell membranes is a required step in viral infection, as this releases the viral core into the host cytoplasm. The viral envelope is enriched in cholesterol compared to the apical membrane of cells from which it buds^1^, and cholesterol has been shown to enhance the efficiency and rate of fusion in model systems^2^,^3^, hemagglutinin-expressing cells^4^,^5^, and between live virions and synthetic liposomes^6^,^7^,^8^. However, the chemical details of how cholesterol promotes fusion remain unknown. To help differentiate among mechanistic hypotheses for the role of cholesterol in influenza viral fusion, we have systematically exchanged cholesterol for each of a set of alternate sterols and measured the rate and efficiency of fusion between X-31 influenza virions and target liposomes.

Several hypotheses have been advanced for how cholesterol may promote influenza viral fusion. Among these are that cholesterol may stabilize highly curved lipid intermediates during fusion^4^, that it may stabilize fusion pores^4^,^5^, that it may affect hemagglutinin spatial localization^9^,^10^,^11^,^12^,^13^, and that it may alter membrane phase behavior or ordering in a way that promotes fusion^14^,^15^. It has also been shown to reduce contents leakage during viral fusion^2^ and may modulate leaky versus non-leaky fusion pathways^16^. Since many of these mechanisms are difficult to observe directly under the conditions of fusion, there has been considerable debate in the field regarding either the existence or the relative functional contributions of these different possible roles for cholesterol.

Since cholesterol can alter membrane spatial organization, it can potentially alter both viral binding to target receptors and the fusion process itself. Because these two processes can be difficult to disentangle in bulk fusion assays, we used a fusion assay without glycan receptors in the target membranes^7^. We have previously shown that fusion proceeds similarly in this bulk assay with and without Gd1a receptors, although at a somewhat faster rate with receptors present. We therefore believe that for this study of sterol effects on fusion, ruling out a sterol effect on hemagglutinin engagement by receptor is desirable.

The panel of sterols tested was selected to probe chemical effects on liquid-liquid phase coexistence, membrane bending rigidity, and polar substitutions at the terminal hydroxyl (Fig. 1). Liquid-liquid phase coexistence has been viewed as related to cholesterol’s ability to promote heterogeneous lateral organization in biological membranes, although the precise mechanism remains under debate^17^,^18^,^19^,^20^,^21^.

A number of sterols shown to permit liquid-liquid phase coexistence^22^,^23^ were tested: epicholesterol, dihydrocholesterol, and ergosterol. In addition, coprostanol and cholestenone were selected as sterols that permit solid-liquid phase coexistence at low temperatures but not liquid-liquid phase coexistence under conditions where cholesterol-containing mixtures form such regions readily, and lanosterol was selected as a sterol that does not readily accommodate phase coexistence^22^,^24^,^25^,^26^,^27^. Sterols with polar substitutions were selected as cholesterol sulfate (negatively charged at fusion pH), cholesteryl dimethyl-aminoethyl carbamate, and cholesteryl trimethyl-aminoethyl carbamate (both positively charged at fusion pH; abbreviated dimethyl carbamate and trimethyl carbamate in figure legends). Data on how different sterols perturb bending moduli are less comprehensive, but several analyses yield the following series for the elastic bending modulus *k*_*c*_: cholesterol sulfate ~ cholesterol > lanosterol > ergosterol^28^,^29^,^30^. This panel of sterols thus independently varies support for liquid-liquid phase coexistence, changes to elastic bending moduli, and headgroup polarity to examine their effects on influenza viral fusion.

## Materials and Methods

### Sterols

Cholesterol, ergosterol, dihydrocholesterol, coprostanol, and cholesteryl sulfate were purchased from Sigma (St. Louis, MO), epicholesterol from Steraloids, cholestenone and lanosterol from Avanti Polar Lipids, cholesteryl N-(2-dimethylaminoethyl)carbamate and cholesteryl N-(trimethyl-ammonioethyl) carbamate chloride from Santa Cruz Biotechnology. Sterol stocks were prepared at 20 mM in chloroform, except for cholesteryl sulfate which was prepared in methanol, stored at −20 °C and pre-warmed to 25 °C prior to use. Methyl-β-cyclodextrin (MβCD)-sterol complexes were formed in phosphate buffered saline (10 mM phosphate/90 mM citrate/150 mM NaCl, pH 7.4; PBS-citrate) at a molar ratio of 1:2. Sterol suspensions were sonicated for 30 s and incubated at 37 °C overnight, at which point any insoluble sterol was removed via 0.2 μm filtration.

### Influenza virus

Egg-grown influenza A X-31, A/Aichi/68 (H3N2) was purchased from Charles River Laboratories. Cholesterol extraction was performed as previously described^7^ via incubation of virus with 20 mM MβCD for 30 min at 37 °C. Virus was then re-isolated from MβCD-cholesterol complexes via centrifugation at 4 °C, 14000 rpm/20187 rcf for 40 min, re-suspended in PBS-citrate pH 7.4 and incubated at 4 °C overnight. Re-addition of cholesterol or other sterols was performed by mixing cholesterol-depleted virus with MβCD-sterol complex and PBS-citrate pH 7.4 at a volumetric ratio of 2:1:5, resulting in final concentrations of approximately 0.5 mg/mL viral protein and 1.7 mM MβCD, and incubating at 37 °C for 30 min, after which time virus was re-isolated as described above. Protein concentration in viral samples was measured by a Bradford assay (Bio-Rad Laboratories, Hercules, CA) and cholesterol concentration using an Amplex Red cholesterol oxidase assay (Life Technologies, Frederick, MD). Phospholipid content was measured using a standard phosphate assay as detailed previously^7^. Quantitation of sterols in viral lipid extracts (produced using chloroform/methanol-HCl extraction at a 1:2:0.8 v/v/v ratio as detailed previously^31^) was performed by the Kansas Lipidomics Research Center via GC/MS for non-polar sterols and electrospray ionization mass spectrometry for polar sterols. Molar amounts of non-polar sterols were standardized using a standard curve of known sterol concentrations using the same GC/MS protocol performed at the Stanford University Department of Chemistry.

### Target liposomes

Unlabeled and dye-conjugated phospholipids were purchased from Avanti Polar Lipids and soluble ANTS and DPX from Life Technologies. Large unilamellar vesicles (LUVs) were extruded through 100 nm pores from a mixture of 30 mol% POPE, 1.5 mol% NBD-DOPE, 1.5 mol% Rh-DOPE, 47 mol% POPC, and 20 mol% sterol. Liposomes were formed to encapsulate 12.5 mM ANTS and 45 mM DPX in PBS-citrate pH 7.4 as previously described^7^. Vesicles were isolated from free ANTS/DPX molecules by size-exclusion chromatography.

### Fusion kinetics

The fluorescence dequenching assay we have described previously^7^ was used to measure lipid and contents mixing kinetics. Briefly, lipid mixing was measured as dequenching of the NBD/Rh FRET pair incorporated in liposomal membranes. Contents mixing was measured as dequenching of the soluble ANTS/DPX complex encapsulated in liposomes; excess DPX in the external buffer serves to suppress any dequenching from contents leakage such that >90% of the dequenching signal results from contents mixing. Fusion was triggered via addition of 2 M citric acid to adjust the pH to 5.0, and fluorescence was measured excitation/emission wavelengths of 460/538 nm and 360/530 nm for NBD-Rh and ANTS-DPX dequenching respectively. Lipid mixing values were normalized as previously described: I_norm_ = (I_obs_ − I_0_)/(I_max_ − I_0_), where Imax is the fluorescence observed after addition of Triton X-100 to a final concentration of 1%. Contents mixing values were reported as fractional increase over baseline. Fusion rates were determined via single-exponential fits with a phase-shift parameter: I_norm_(t) = *a* * (1 − exp(−*b* *(t + t_0_))). As we have described previously^7^, lipid and contents mixing in this regime fit well to a single-exponential curve with a plateau. The time constant for the exponential curve yields an aggregate rate of mixing, and the plateau yields relative efficiency.

### Growth of cholesterol-depleted influenza virus

Madin-Darby canine kidney (MDCK) cells were maintained in DMEM + 10% FBS. Lovastatin at 4 μM or control carrier (0.04% v/v DMSO) was added for 24 h prior to infection. For the infection, 95% confluent cells in a 75 cm^2^ flask were washed with PBS, and 15 ml of infection media was added to the cells: influenza virus at 0.01 multiplicity of infection in DMEM with 0.01% FBS and 2 μg/ml trypsin with 4 μM lovastatin or DMSO control as indicated. Supernatant was harvested after 48 h at 37 °C, and virus was isolated on a 25% sucrose cushion in Tris-EDTA buffer (10 mM Tris/100 mM NaCl/1 mM EDTA, pH 7.4) via ultracentrifugation at 4 °C, 28000 rpm/58568 rcf for 2 h. Virus was then resuspended and dialyzed in PBS with 1 mM EDTA using a dialysis membrane with MWCO 6–8,000 at 4 °C overnight, after which it was concentrated by centrifugation at 4 °C, 14000 rpm/20187 rcf for 40 min and resuspended in PBS-citrate.

### Electron cryomicroscopy

Electron cryomicroscopy and analysis was performed as detailed in prior work^32^. Briefly, 3 microliters of sample were applied to a carbon-coated grid (Cat. No. 2/2-4C C-flats; ProtoChips, Raleigh, NC), blotted with filter paper, and plunge-frozen in liquid ethane. Samples were imaged in a Tencai F20 Twin transmission electron microscope (FEI, Hillsboro, OR) at −180 C with a nominal defocus of 3 microns and a magnification of 29,000x, operating at 120 kV. Images were recorded on a 4096 × 4096 pixel CCD, such that each pixel represents 3.7 Å. Virion interiors were manually boxed using the EMAN2 software^33^ using a fixed box size of 158 × 158 pixels. Radially averaged 2D Fourier transforms were computed for at least 290 virions for each sample condition using code freely available at https://github.com/kassonlab/em-spatial-analysis. These spectra approximate the radial distribution function for electron density in a 2D projection of the virion.

## Results

We tested how sterol chemical properties influence viral membrane fusion by measuring the rates and efficiencies of influenza virions fusing to synthetic liposomes. Three sets of perturbations were tested: 1) influenza virus was produced in the presence of the cholesterol synthesis inhibitor lovastatin and compared to unmodified egg-grown virus 2) unmodified virus was fused to liposomes containing 20 mol% of different sterols, and 3) different sterols were added to the envelope of influenza virions that had been previously depleted of cholesterol; these “sterol-swapped” virions were then fused to liposomes containing 20 mol% cholesterol. The precise sterol concentration of target endosomes at the time of fusion is not known, and 20 mol% sterol was chosen as an arbitrary composition that yields well-behaved liposomes for a large number of sterols. In all cases, fusion was monitored using the fluorescence dequenching assay we have described previously^7^: fusion between virions and liposomes was triggered by lowering the pH to 5.0, and bulk dequenching kinetics of lipid and contents dyes in the liposomes were used to monitor fusion. Results of these assays are detailed below.

**Figure 2:**
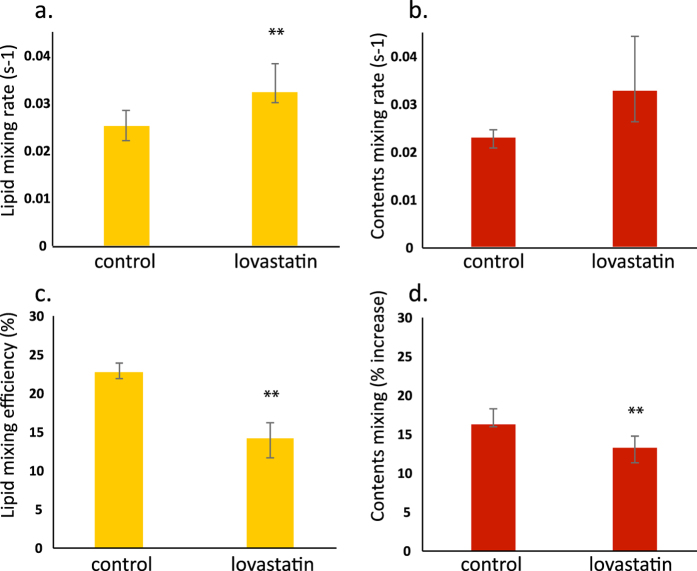
Depletion of cholesterol reduces fusion efficiency and increases fusion rates in cultured virus. X-31 influenza virus was grown in MDCK cells in the presence of carrier control or 4 μM lovastatin. Lovastatin decreased the envelope cholesterol content, increased the fusion rate (panels a and b), and decreased the fusion efficiency (panels c and d) of the isolated virus. This decrease in efficiency was fully reversible via addition of exogenous cholesterol to the viral envelope using MβCD. Bars represent the median of 5–8 experiments per condition, while error bars show the inter-quartile range. Statistical significance was assessed via the Kolmogorov-Smirnov test, with ** marking significant differences, p < 0.05.

Growing X-31 influenza virus in the presence of the HMG-CoA reductase inhibitor lovastatin yielded similar effects on viral fusion kinetics to those we have previously shown from chemical depletion of cholesterol from already-produced influenza virions. Virus propagated in MDCK cells in the presence of 4 μM lovastatin had a reduced level of cholesterol in its envelope: 0.16:1 cholesterol:phospholipid ratio as opposed to 0.38:1 in virus that was similarly propagated in the absence of lovastatin and 0.77:1 in virus that had been grown in eggs. The decrease in fusion efficiency and increase in fusion rate (Fig. 2) were comparable to that of virus chemically depleted of cholesterol using methyl-beta-cyclodextrin (MβCD), although much milder than treatment with 20 mM MβCD, which produced a cholesterol:phospholipid ratio of 0.01–0.02:1. X-31 virus that was maintained in eggs until infection of MDCK cells had a lower cholesterol:phospholipid ratio than either egg-grown virus or H7N1 virus previously reported using MDCK cells^1^; this may represent a combination of strain and host differences, an adaption of envelope composition that occurs only on serial passage, or other as-yet-unidentified factors.

**Figure 3:**
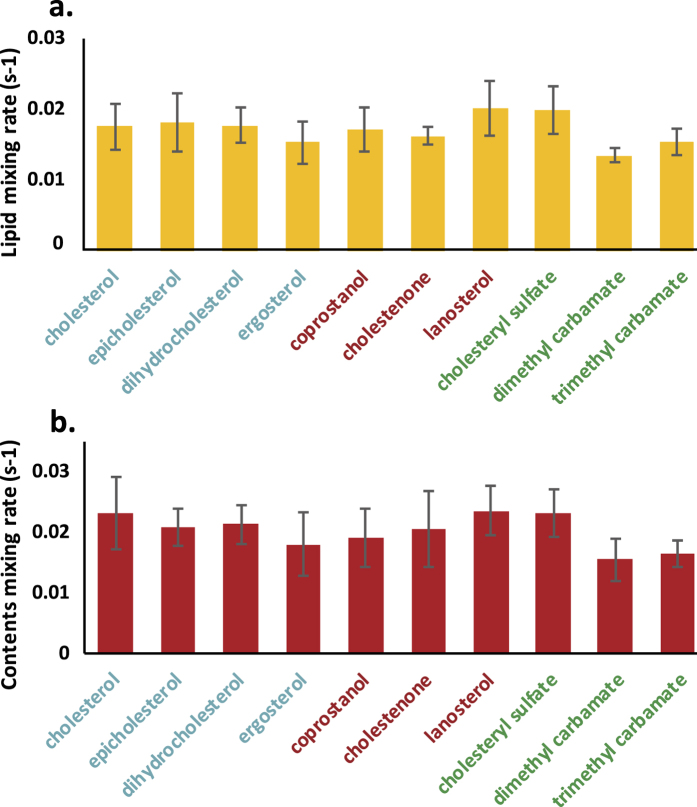
Influenza fusion rates are insensitive to sterol identity in target liposomes. Influenza viral fusion was measured to target liposomes containing 20 mol% of the designated sterol. Bars show mean rate across 4–8 experiments per condition, while error bars show the standard deviation. No statistically significant differences (assessed via Kolmogorov-Smirnov tests with Bonferroni multiple-hypothesis correction) were observed in fusion rates across sterols tested. Sterol names are colored to match the categories of membrane effects summarized in Fig. 1.

Surprisingly, influenza viral fusion to liposomes was almost completely insensitive to the chemical identity of the sterol present in the liposomes. Previous data have shown a strong dependence of both fusion rate^7^ and efficiency^6^,^7^ on the cholesterol mol% present in target liposomes. The cholesterol dependence of fusion to cellular membranes may depend on several factors, including the bending energy required to form highly curved fusion intermediates, cholesterol-mediated spatial heterogeneity within the membrane, or on more direct peptide-sterol interactions. We therefore tested sterols shown to modulate phase behavior and domain formation in model mixtures (including coprostanol and cholestenone), ones shown to alter membrane bending moduli (ergosterol and lanosterol), as well as others. As shown in Fig. 3 and S1, none of these significantly altered fusion rates or efficiencies (Kolmogorov-Smirnov test with Bonferroni multiple-hypothesis correction; p > 0.05 for a single-tailed distribution). It is important to note that substantial lateral membrane heterogeneity or co-existing fluid phases would not be expected in target liposomes of the composition used: POPC:POPE:sterol—this was a deliberate choice to use one simple mixture for the liposomes and a more complex physiological mixture in the viral envelope.

**Figure 4:**
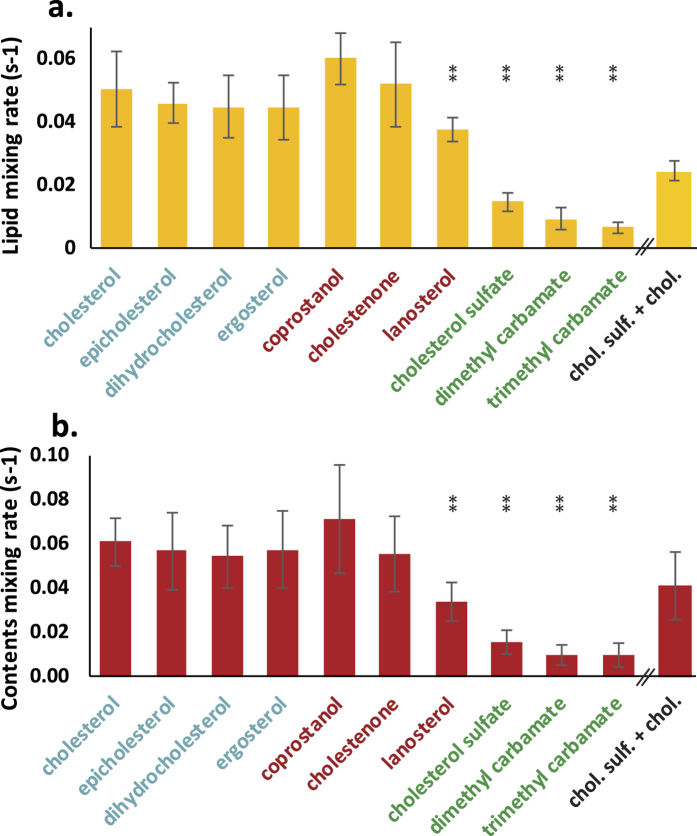
Influenza fusion rates are decreased moderately by lanosterol and greatly by polar sterols added to the viral envelope. Fusion rates were measured between liposomes and influenza virus that had cholesterol replaced with the designated sterol using first depletion via MβCD and then addition of MβCD-sterol complex. Substitution of non-polar sterols did not significantly alter viral fusion kinetics compared to cholesterol, with the exception of lanosterol, which showed a slight but significant slowing. The polar sterols cholesterol sulfate, cholesteryl dimethyl-aminoethyl carbamate, and cholesteryl trimethyl-aminoethyl carbamate all caused significant slowing of fusion. Addition of a mixture of cholesterol sulfate and cholesterol was able to partially rescue the cholesterol sulfate phenotype. Bars show the mean rate across 6–14 experiments per condition, while error bars show the inter-quartile range. Statistical significance was assessed via the Kolmogorov-Smirnov test with Bonferroni correction. Sterol names are colored to match the categories of membrane effects summarized in Fig. 1.

Fusion rates and efficiencies were also surprisingly robust to perturbing the chemical identity of sterols in the influenza viral envelope, but some significant differences were observed compared to the same panel of sterols in target liposomes. Among nonpolar sterols, lanosterol was the only one that significantly slowed fusion (Fig. 4, fusion efficiencies in Fig. S2): both lipid and contents mixing were significantly slower via a Kolmogorov-Smirnov test with Bonferroni correction (p-values 0.001 and 1.6 × 10^−4^ respectively). The most striking differences were observed with the polar sterols cholesterol sulfate (negative charge at the pH of fusion) and dimethyl-aminoethyl cholesteryl carbamate and trimethyl-aminoethyl cholesteryl carbamate (both positively charged at the pH of fusion). Virus containing these sterols showed moderately reduced fusion efficiencies but greatly reduced rates of fusion: lipid and contents mixing rates decreased by a factor of 3–8, p-values were all <3 × 10^−6^ as assessed by Kolmogov-Smirnov tests with Bonferroni correction. Efficiency of sterol delivery to the envelope was assessed by mass spectrometry on lipid extracts from each viral sample and is given in Table S1. Although a few sterols were delivered to the viral envelope in substantially lower amounts (ergosterol, cholestenone, and dimethyl-aminoethyl cholesteryl carbamate), at least one sterol in each chemical “series”—sterols supporting liquid-liquid phase coexistence, sterols not supporting multiple liquid phases, sterols altering membrane bending moduli, and polar sterols—delivered with high efficiency to the viral envelope. Therefore the fundamental result regarding sterol chemical identity appears robust. In addition, mass spectrometry of lipid extracts from viral samples is a bulk assay that may include non-viral membrane particles that co-purify with influenza virus. Although specifically measuring sterol concentration in fusion products is not feasible at this time, we have used electron cryo-microscopy of sterol-replaced virions as a virus-specific assay as discussed below.

While transbilayer exchange or “flip-flop” of cholesterol is believed to be relatively fast^34^,^35^,^36^,^37^, particularly in membranes out of equilibrium, polar sterol exchange rates are both likely slower and less extensively characterized experimentally. We therefore predicted transbilayer exchange rates by performing coarse-grained molecular dynamics simulations of planar bilayers containing POPC and 20 mol% sterol in one leaflet only. Based on one 30-microsecond run per sterol, these simulations predict a cholesterol flip-flop rate of 2.0 ± .01 × 10^6^ s^−1^ at 320 K from this asymmetric starting condition and cholesterol sulfate flip-flop rate >6000-fold slower. Cholesterol flip-flop rates are within 25% of those predicted via similar approaches previously^34^. Details of the simulations are given in the Supporting Information and simulation snapshots in Fig. S3. We therefore conclude that the polar sterols likely do not achieve transbilayer equilibrium on the timescale of our fusion experiments.

To test the hypothesis that polar sterols slow fusion due to their inability to equilibrate across the viral membrane, we added a mix of cholesterol sulfate and cholesterol to cholesterol-depleted influenza virus and measured fusion kinetics. If the inhibitory effect of cholesterol sulfate were solely due to its negative charge, addition of cholesterol to a constant amount of cholesterol sulfate would not be expected to increase fusion rates. However, if the decrease in fusion rate results from bilayer asymmetry, addition of a second sterol that can readily repartition across the bilayer should mitigate the effect. As shown in Fig. 4, delivery of a mixture of cholesterol sulfate and cholesterol produced fusion and hemifusion rates that were intermediate between delivery of pure cholesterol sulfate and delivery of pure cholesterol. The amount of cholesterol sulfate delivered was within 10% of that delivered in the pure cholesterol sulfate sample. Although this result does not directly establish a mechanistic effect based on bilayer asymmetry, it is nevertheless strongly suggestive.

**Figure 5:**
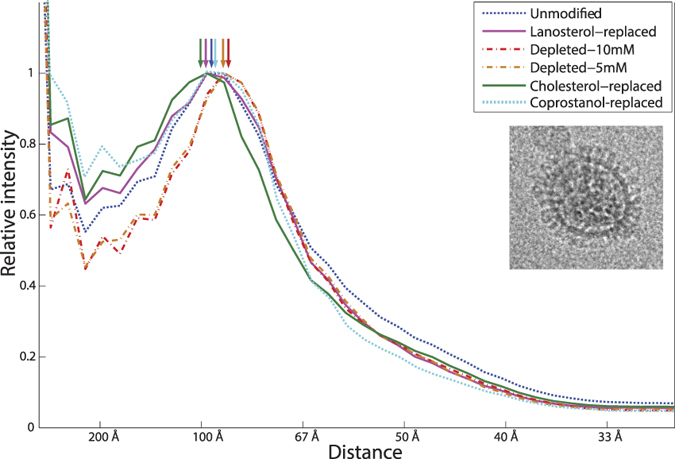
Hemagglutinin spatial distribution in lanosterol-replaced influenza virus is similar to cholesterol-replaced virus but not cholesterol-depleted virus. Hemagglutinin spatial distribution is estimated from radially averaged 2D Fourier transforms of transmission electron micrographs. These approximate a pair correlation function of electron density. Prior experiments using anti-HA monoclonal Fabs^32^ have shown that the HA density can be assigned to the peak at ~100–85 Å, which agrees with previous estimates of influenza surface glycoprotein spacing^41^,^42^. This peak shifts laterally upon cholesterol depletion and back upon replenishment using either cholesterol, lanosterol, or coprostanol. The mode of the peak, indicating typical nearest-neighbor spacing of hemagglutinin on the viral envelope, is identical for cholesterol-replaced, lanosterol-replaced, and coprostanol-replaced virus at 97 Å. This analysis was performed in an identical fashion to our previous work, with difference being the use of sterols other than cholesterol. An electron micrograph of a lanosterol-replaced virion is shown in the inset.

In other recent work, we found that cholesterol could alter the lateral spacing of hemagglutinin on the influenza viral surface, as assessed by electron cryomicroscopy, and that this altered spacing could partially account for changes in fusion kinetics^32^. The model we proposed to explain that finding is that the chemical potential of lipids in the viral envelope “close” to hemagglutinin is much more sensitive to mole fraction cholesterol than is the chemical potential of lipids “far” from hemagglutinin. Since our cryo-EM data probed protein distribution and not lipid distribution in the viral envelope, we could not discriminate between small lipid shells around the hemagglutinin and larger nanodomains that could, in the large-size limit, include much of the viral surface. The findings we report here are consistent with that model but yield additional evidence regarding the spatial scales involved. Since replacing cholesterol with lanosterol in the viral envelope slowed fusion and lanosterol should not support liquid-liquid phase coexistence, we tested whether lanosterol resembled cholesterol in its ability to drive sterol-dependent patterning of hemagglutinin on the viral surface. We performed electron cryo-microscopy of cholesterol-replaced and lanosterol-replaced influenza virions and measured typical hemagglutinin-hemagglutinin nearest neighbor spacings (Fig. 5) in a manner similar to our prior work^32^. We found that lanosterol-replaced virions have similar hemagglutinin spatial distribution to cholesterol-replaced virions and different from sterol-depleted virions. This suggests that the spatial patterning of hemagglutinin on the viral surface, while sterol-dependent, does not depend on cholesterol’s ability to support phase coexistence. Furthermore, since lanosterol-replaced virions show slower rates of fusion yet display the same hemagglutinin spatial patterning as unmodified virions, these data suggest a second component to the dependence of fusion kinetics on cholesterol beyond the spatial patterning of hemagglutinin (which should reflect cholesterol concentration).

Because the mass spectrometry data suggested uneven mean sterol concentration in the sterol-replaced viral samples, we further used spatial patterning of hemagglutinin to probe sterol activity, and hence implicitly sterol delivery, in virions observed via electron cryo-microscopy. We had previously reported a change in the spatial patterning of hemagglutinin upon increasing removal of cholesterol from the viral envelope^32^. Therefore, we hypothesize that a hemagglutinin spatial patterning similar to unmodified virus should indicate good sterol delivery. As shown in Fig. 5, coprostanol also induced a cholesterol-like spatial distribution of hemagglutinin, indicating sufficient delivery to influenza virions.

## Discussion

Our data show that the rate-limiting step for influenza viral fusion is sensitive to sterol quantity but surprisingly robust to sterol chemical structure. Perturbation to fusion rates that we do observe from changing the chemical structure of sterols in the viral envelope do not clearly correlate with the effects that would be expected if the sterol mode of action primarily affected lipid phase behavior or bending moduli. Instead, they most strongly depend on sterol polarity and predicted transbilayer flip-flop rates. Our results therefore suggest a model for cholesterol activity in the rate-limiting step for influenza viral fusion as follows. 1) Fusion rates depend on mole fraction cholesterol in a fashion that may vary depending on the fusion partner being modified and the sterol composition in each membrane leaflet. 2) Asymmetric distribution of sterols in the viral membrane can inhibit fusion; this can be rescued with a sterol that readily exchanges across the membrane. 3) The effect of cholesterol in physiological membrane environments does show some chemical specificity but is not consistent with a model where sterol effects on bending moduli control the rate-limiting step for fusion nor one where properties correlating with macroscopic phase separation would clearly explain the perturbation to fusion rates.

More subtle effects of sterol chemical structure on lateral distribution of proteins and lipids cannot be ruled out and will be the subject of future investigation. In studies of giant unilamellar vesicle model systems, Bacia and co-workers found that cholesterol sulfate in mixture with cholesterol could modify both the size of liquid-ordered domains and the curvature of those domains^38^, although cholesterol sulfate itself induced identical bending elasticity in binary DMPC/sterol mixtures compared to identical mol% cholesterol^30^. It is tempting to speculate whether cholesterol sulfate and potentially the cholesteryl carbamates could have similar effects in altering the lateral organization and curvature of the viral envelope, or whether their effects might be focused on curved lipidic intermediate structures in fusion stalk formation. Such transient structures are of course particularly difficult to study directly. Prior work on Sendai virus has shown that addition of cholesterol sulfate to target membranes can inhibit fusion—there, it was proposed that cholesterol sulfate may stabilize L_α_ bilayers and impede the formation of highly curved fusion intermediates^39^. Although our data do not show an inhibition of influenza fusion by 20 mol% cholesterol sulfate in target liposomes, only in the viral envelope, the Sendai virus data do suggest a potential role for cholesterol sulfate in modulating free energies of fusion intermediate formation. Similarly, different sterols may modify the intrinsic curvature of inner and outer leaflets throughout the fusion process, and combinations of different sterol distribution between leaflets and different curvature effects between sterols may account for some of the differences observed, as has previously been demonstrated for other curvature perturbations^40^. Since all of the sterols studied here except cholesterol sulfate and the carbamates redistribute rapidly between bilayer leaflets and we lack a good experimental assay for trans-leaflet distribution, we are hesitant to make particular mechanistic inferences regarding differential curvature modulation in the two leaflets. It however remains a candidate explanation for the different effects observed here.

**Figure 6:**
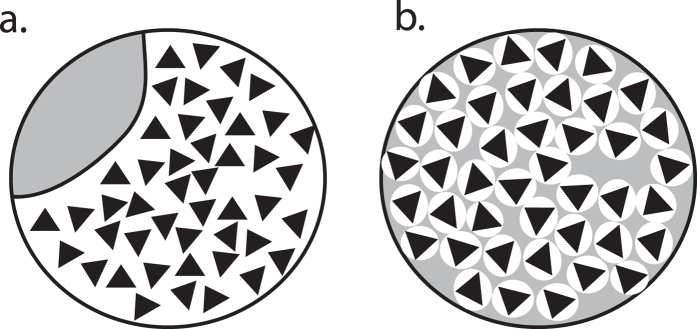
Models for lipid spatial organization in the influenza viral envelope. If hemagglutinin undergoes sterol-dependent changes to its spatial organization in influenza virions, two extreme models are schematized: a large sterol-dependent “patch” (panel a, white with black triangles representing hemagglutinin) or small sterol-dependent “shells” (panel b). Since model (**a**) would likely depend on liquid-liquid phase separation and since our electron microscopy data show that lanosterol can induce similar spatial changes to cholesterol, we find model (**b**) more probable.

Although current techniques do not permit direct observation of nanoscale lipid patterning on the viral surface, we are now able to make several inferences regarding lipid spatial distribution and its effect on the rate-limiting step of viral fusion in this system. Because the argument for large cholesterol-dependent nanodomains in the viral envelope rests on notions of liquid-liquid phase separation or its nanoscopic correlates, our observation that influenza fusion is robust to cholesterol/coprostanol exchange suggests that spatial patterning relevant to fusion rates is not connected to liquid-liquid phase coexistence (which coprostanol does not support) and is thus more likely to involve local interactions than larger-scale separation. A model for cholesterol activity in the influenza viral membrane must thus explain two observed phenomena: first, that cholesterol depletion reversibly draws hemagglutinin trimers closer together and second, that cholesterol can be readily substituted for sterols that do not support liquid-liquid phase separation without altering the rate-limiting step of fusion. The simplest explanation is that the lipid context relevant to hemagglutinin patterning and fusion kinetics is very local—more resembling a “shell” around each hemagglutinin than a large “patch” occupying much of the envelope (Fig. 6). Our finding that lanosterol-replaced virions have hemagglutinin spatial distribution similar to cholesterol-containing virions yet different from cholesterol-depleted ones suggests that the cholesterol effect on hemagglutinin organization is not linked to liquid-liquid phase coexistence. It thus adds further weight to the “shell” model where the presence of sterol affects hemagglutinin lateral organization (and thus has an indirect effect on fusion kinetics), whereas the identity of certain sterols such as lanosterol can affect hemagglutinin activity in a local manner. Whether the sterols in this shell affect the rate-limiting step of influenza membrane fusion by altering local membrane properties or via direct interaction with hemagglutinin remains unknown.

## Additional Information

**How to cite this article**: Zawada, K. E. *et al*. Influenza viral membrane fusion is sensitive to sterol concentration but surprisingly robust to sterol chemical identity. *Sci. Rep.* **6**, 29842; doi: 10.1038/srep29842 (2016).

## Supplementary Material

Supplementary Information

## Figures and Tables

**Figure 1:**
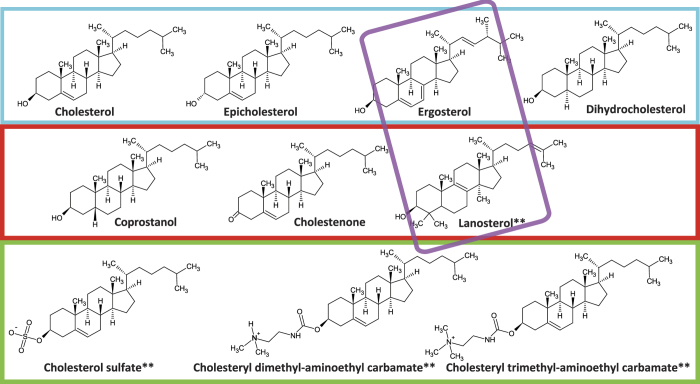
Chemical structures of the sterols used in this study. Ionization states shown are the predominant forms at both pH 7.4 and the fusion pH of 5.0. Colored boxes denote groups of sterols that support liquid-liquid phase coexistence (cyan), those that do not (red), polar sterols (green), and sterols with lower elastic bending moduli (purple). Sterols that cause a significant change in fusion rates when incorporated into the viral membrane are marked by **.
