# Supplementary material for: Influenza viral membrane fusion is sensitive to sterol concentration but surprisingly robust to sterol chemical identity
Source: Sci Rep. 2016 Jul 19;6:29842. doi: 10.1038/srep29842 (PMC4949436; doi:10.1038/srep29842)
Supplement: Supplementary Information [file srep29842-s1.pdf]

Supporting Information for

Influenza viral membrane fusion is sensitive to sterol concentration but surprisingly robust to sterol chemical identity

Katarzyna E. Zawada, Dominik Wrona†, Robert J. Rawle, and Peter M. Kasson*

Departments of Molecular Physiology and Biological Physics and of Biomedical Engineering, University of Virginia. Charlottesville, VA 22908. †Present Address: Division of Immunology, University Children's Hospital Zurich and Children's Research Centre, Zurich, Switzerland.

*To whom correspondence should be addressed. E-mail: kasson@virginia.edu

Supplemental Methods:

Coarse-grained molecular dynamics simulations were performed using Gromacs 4.6(1) and the MARTINI v2 coarse-grained force field(2,3). Initial POPC:Chol coordinates were prepared using the CHARMM-GUI membrane builder(4) and coarse-grained using the lipid-converter tool(5). Cholesterol was converted manually to cholesterol sulfate in coarse-grained simulations via a simple bead swap of the ROH group to an SO3- group. A 30-fs timestep was employed with standard MARTINI v2 parameter settings except as noted. These standard settings include van der Waals and coulombic forces shifted to zero at 1.2 nm. Temperature was maintained at 320 K using the velocity-rescaling thermostat(6), and pressure was maintained at 1 bar in a semi-isotropic fashion using the Parrinello-Rahman barostat. Coordinates were written at 7.5 ns intervals, and sterol flip-flop was assessed using analyze_cholflip.py, code available from www.github.com/kassonlab/sterol_analysis. Error analysis was performed using bootstrap resampling over sterol molecules. Coarse-grained simulation times were multiplied by a factor of 4 as a rough time-normalization for lateral diffusion as has been done in previous analyses of cholesterol flip-flop(7).

**sample**	**chole sterol**	**epi-choles terol**	**dihydro choleste rol**	**ergo-sterol**	**copros tanol**	**cholest enone**	**lanos terol**	**choleste rol sulfate**	**dimethyl carbama te**	**trimethyl carbamate**
**chol. (nmol/50 uL)**	16.0	0.10	0.095	0.087	0.11	0.073	0.091	*	*	*
**analogue (nmol/50 uL)**	0	3.81	15.1	1.46	7.96	1.39	3.03	3.06	0.918	19.2
**protein (mg/mL)**	0.60	0.63	0.42	0.58	0.77	0.63	0.54	0.56	0.99	0.52

Table S1. Efficiency of sterol delivery to influenza virus using MβCD as a carrier. Sterol and protein concentrations in sterol-substituted X-31 influenza virus are listed

above. Cholesterol and cholesterol analogue concentrations were determined by mass spectrometry, and protein concentration was determined via Bradford assays. Polar sterols were assayed via electrospray mass spectrometry, which did not permit simultaneous measurement of cholesterol content.

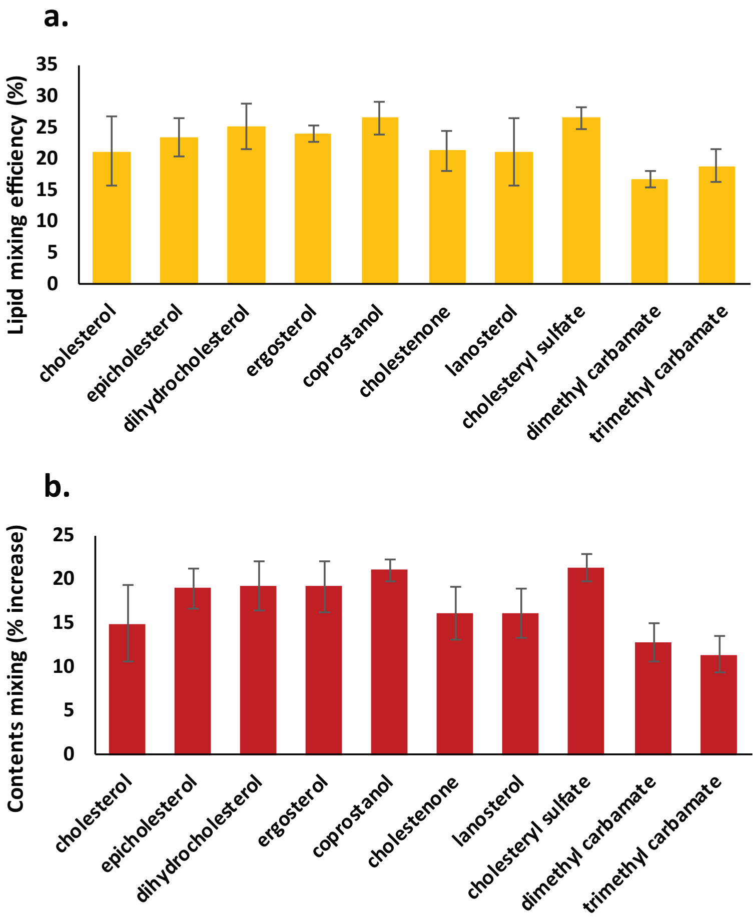

Figure S1. Fusion efficiency of X-31 influenza virus to liposomes containing different sterols. Target liposomes were created via extrusion containing 20 mol % of the indicated sterol. Bars show mean rate across 4-8 experiments per condition, while error bars show the standard deviation. Panel (a) plots lipid mixing, while panel (b) plots contents mixing.

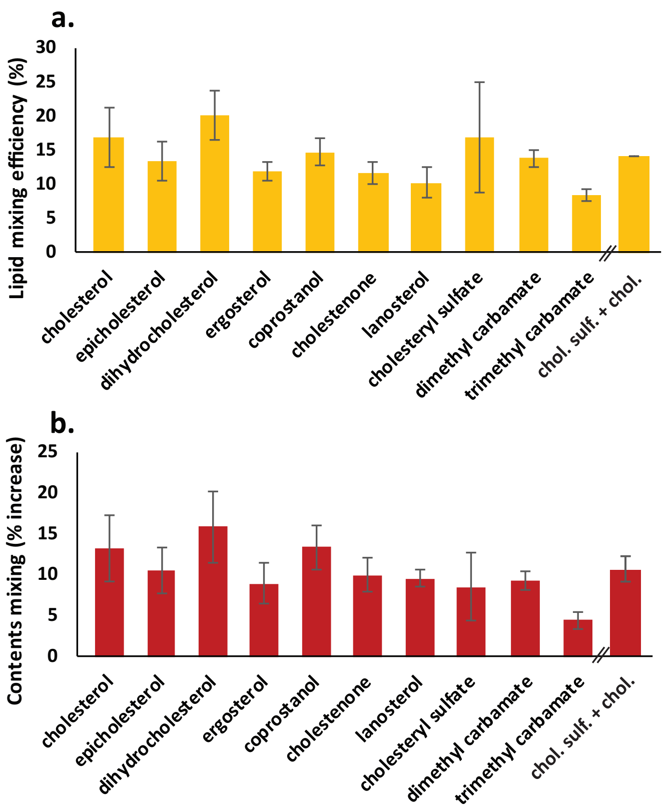

Figure S2. Fusion efficiency of sterol-substituted X-31 influenza virus to liposomes containing 20 mol % cholesterol. Fusion was measured between liposomes and influenza virus that had cholesterol replaced with the designated sterol using first depletion via MβCD and then addition of MβCD-sterol complex. Bars show the mean rate across 6-14 experiments per condition, while error bars show the inter-quartile range.

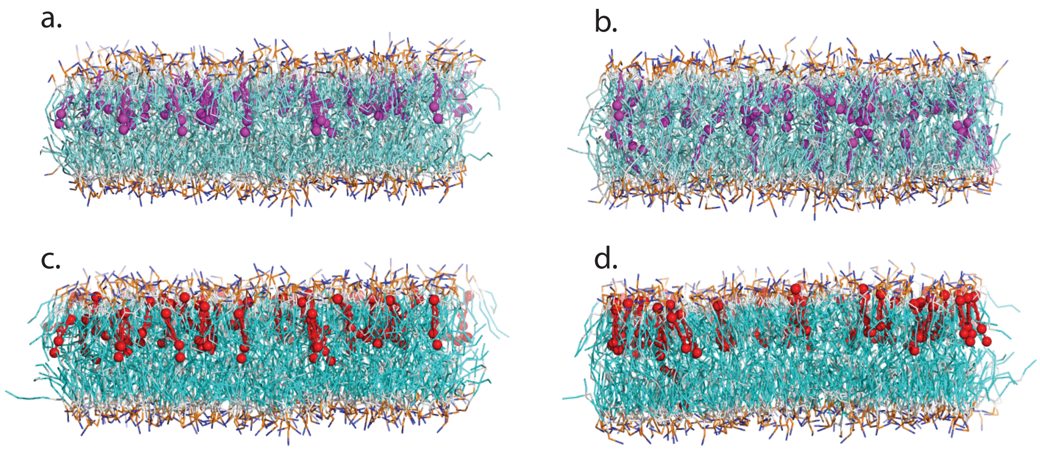

Figure S3. Coarse-grained molecular dynamics simulations of planar bilayers composed of POPC with 20 mol % sterol added to one leaflet only. Panels (a) and (b) show simulations of cholesterol, while panels (c) and (d) show cholesterol sulfate. Panels (a) and (c) depict snapshots at simulation start, while panels (b) and (d) depict snapshots after 30 microseconds of simulation. Cholesterol is shown in magenta, while cholesterol sulfate is shown in red. Solvent is not rendered.

References cited

1. Pronk, S., Pall, S., Schulz, R., Larsson, P., Bjelkmar, P., Apostolov, R., Shirts, M. R., Smith, J. C., Kasson, P. M., van der Spoel, D., Hess, B., and Lindahl, E. (2013) GROMACS 4.5: a high-throughput and highly parallel open source molecular simulation toolkit. *Bioinformatics* **29**, 845-854
2. Marrink, S. J., Risselada, H. J., Yefimov, S., Tieleman, D. P., and de Vries, A. H. (2007) The MARTINI force field: coarse grained model for biomolecular simulations. *J Phys Chem B* **111**, 7812-7824
3. Marrink, S. J., de Vries, A. H., Harroun, T. A., Katsaras, J., and Wassall, S. R. (2008) Cholesterol shows preference for the interior of polyunsaturated lipid membranes. *J Am Chem Soc* **130**, 10-11
4. Jo, S., Lim, J. B., Klauda, J. B., and Im, W. (2009) CHARMM-GUI Membrane Builder for mixed bilayers and its application to yeast membranes. *Biophys J* **97**, 50-58
5. Larsson, P., and Kasson, P. M. (2014) Lipid converter, a framework for lipid manipulations in molecular dynamics simulations. *J Membr Biol* **247**, 1137-1140
6. Bussi, G., Donadio, D., and Parrinello, M. (2007) Canonical sampling through velocity rescaling. *J Chem Phys* **126**, 014101
7. Bennett, W. F., MacCallum, J. L., Hinner, M. J., Marrink, S. J., and Tieleman, D. P. (2009) Molecular view of cholesterol flip-flop and chemical potential in different membrane environments. *J Am Chem Soc* **131**, 12714-12720
